# Soret-effect-induced thermoelectrics: confronting the efficiency bottleneck

**DOI:** 10.1093/nsr/nwag138

**Published:** 2026-03-09

**Authors:** Dan Zhao, Qinglin Jiang, Reverant Crispin

**Affiliations:** Laboratory of Organic Electronics, Department of Science and Technology, Linköping University, Sweden; Institute of Polymer Optoelectronic Materials and Devices, Guangdong Basic Research Center of Excellence for Energy and Information Polymer Materials, State Key Laboratory of Luminescent Materials and Devices, South China University of Technology, China; Guangdong Provincial Key Laboratory of Luminescence from Molecular Aggregates (South China University of Technology), China; Laboratory of Organic Electronics, Department of Science and Technology, Linköping University, Sweden

**Figure 1.**
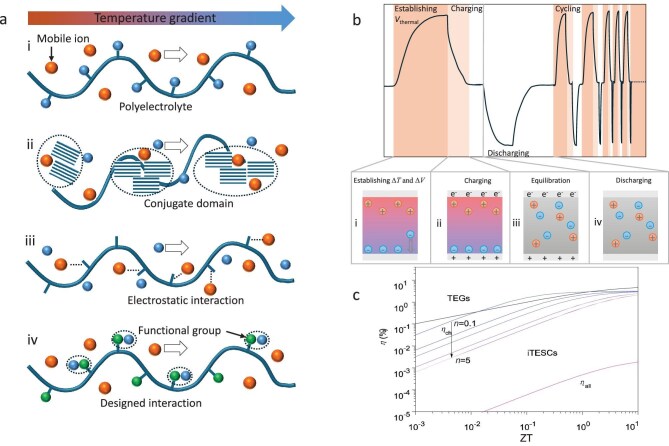
(a) Illustration of typical structures of polymer-based ionic thermoelectric materials. (b) Operation principle and efficiency potential bottleneck for ionic thermoelectric devices (i) establishing Δ*T* and Δ*V* in an open circuit, (ii) connecting the hot and cold electrodes to charge the supercapacitor, (iii) disconnecting the circuit and removing Δ*T* to relax ions in the electrolyte, and (iv) connecting the electrodes for discharge. (c) The dependence of the efficiency of thermoelectric generators (TEGs) and ionic thermoelectric supercapacitors (iTESCs) on the *ZT* value. Black line indicates the efficiency for TEGs, colored lines in the middle show the charging efficiency of iTESCs with different charging completeness (different *n*) and the red line at the bottom shows the overall efficiency of the iTESCs considering the input heat of the whole cycle.

The thermodiffusion of electronic charge carriers in metals and semiconductors has been utilized for over a century to convert heat into electricity through thermoelectric generators (TEGs). However, the conversion efficiency is limited due to the inherently low Seebeck coefficients—typically only 100–200 μV/K. A similar mechanism occurs in electrolytes: ions thermodiffuse under temperature difference (Δ*T*) due to the Soret effect, driven by entropy differences that are often described in terms of Eastman’s dimensionless ‘entropy of transfer’ [1]. The connection to thermoelectric energy conversion emerges when anions and cations thermodiffuse at different rates, leading to charge separation that gives rise to the measured electric potential difference (Δ*V*), manifesting as the ionic Seebeck effect. In 2016, ionic Seebeck coefficients (*α*_i_, defined as the ratio between the measured thermal voltage and the applied temperature difference) exceeding 10 mV/K were reported for a polymer-based electrolyte [2], which is nearly two orders of magnitude higher than those of electronic materials. This breakthrough ignited intense activity, with subsequent reports reaching 20–30 mV/K [3]. Over the past decade, polymer-based ionic thermoelectric materials have evolved from simple polyelectrolytes to systems with deliberately designed structures that enhance the asymmetry in thermodiffusion between cations and anions. As illustrated in Fig. 1a, the thermoelectric potential in polyelectrolytes (i) primarily originates from the size disparity between cations and anions, which leads to distinct ionic mobilities under a temperature gradient. In parallel, hybrid systems based on conducting polymers (ii) have been developed, in which interactions between conjugated polymer domains and doping ions are exploited to amplify the thermally induced ionic potential. In 2019, ionic thermoelectric effects were further demonstrated in ionic liquid-based polymer gels (iii), with the underlying mechanism attributed to electrostatic interactions between mobile ions and dipolar moieties that affect the diffusion coefficient. Since then, the research focus has largely shifted toward gel-based electrolytes, in which tailored hydrogen-bonding or coordination interactions are introduced to modulate ionic thermodiffusion and enhance thermoelectric performance [4]. However, beneath this apparent prosperity lurks a sobering reality: despite remarkable material advances, the field confronts a critical efficiency bottleneck of overall practical efficiency, which threatens to confine Soret-induced thermoelectrics to laboratory curiosities rather than applications in real context.

The fundamental challenge arises from the intrinsic physics of ionic charge transport. Ionic motion in electrolytes is inherently much slower than electronic transport in solids and, critically, ions cannot traverse an external circuit in the same way as electrons do in conventional TEGs. Instead, thermodiffused ions accumulate at the electrode–electrolyte interfaces, forming electric double layers rather than producing a continuous current. As a result, these systems operate as ionic thermoelectric supercapacitors (iTESCs, Fig. 1b): (i) a thermal voltage develops and (ii) charges the devices through an external circuit during heating. For an ideal non-redox system, stored charge remains in the electrodes as long as the thermodiffused ions remain under the temperature gradient. Hence, (iii) subsequent cooling is required to relax the ion distribution before (iv) the stored electrical energy can be consumed [5]. This charge–discharge cycle is inseparable from the temperature cycle, meaning that iTESCs are only suitable for applications involving repeated thermal oscillations, rather than steady-state heat harvesting.

During the heating half-cycle (Fig. 1b(i) and (ii)), the electrolyte absorbs heat (*Q*_m_) to establish Δ*T* between the electrodes. At the same time, ions begin to thermodiffuse and generate Δ*V*. The characteristic time required for this ionic redistribution (*τ*_i_) is governed by the electrode separation (*L*) and ionic diffusion coefficient (*D*), scaling as *τ*_i_ ≈ *L*^2^/*D*. Throughout this period, thermal energy (*Q*_th_) is consumed to sustain Δ*T*. When the circuit is closed, electrons flow through the external load and the electrodes charge accordingly. Importantly, ionic thermodiffusion is typically slower than the electronic charging process, so the overall charging dynamics are limited by ion transport within the electrolyte.

The early attempt to understand the efficiency of iTESCs focused only on the charging process, in which external current is generated, and neglected *Q*_m_ and *Q*_th_. The charging efficiency *η*_ch_ was derived from the ratio between the generated electric energy (*E*_ch_) and the consumed heat (*Q*_ch_) during the charging process. *η*_ch_ can be expressed as a function of the ionic thermoelectric figure of merit *ZT*_i_ = $\frac{{{\sigma }_{\mathrm{i}}\alpha _{\mathrm{i}}^2}}{\lambda }$, where ${\sigma }_{\mathrm{i}}$ is the ionic conductivity, ${\alpha }_{\mathrm{i}}$ is the ionic Seebeck coefficient and $\lambda $ is the thermal conductivity of the electrolyte:


(1) \begin{eqnarray*}
\eta_ {{\mathrm{ch}}} = \frac{{{\mathrm{\Delta }}T}}{{{T}_{\mathrm{H}}}}\frac{{Z{T}_{\mathrm{i}}}}{{2Z{T}_{\mathrm{i}} + \frac{{2nT}}{{{T}_{\mathrm{H}}}} - \frac{1}{2}Z{T}_{\mathrm{i}}\frac{{{\mathrm{\Delta }}T}}{{{T}_H}}}}.
\end{eqnarray*}

The definition of *ZT*_i_ is convenient for comparing ionic thermoelectric materials; however, it is fundamentally different from *ZT* for TEGs because they correlate with the efficiency of different devices. The correlation between *ZT*_i_ and *η*_ch_ depends on an additional parameter related to the charging time: $n = \frac{t}{\tau }$ (*τ* is the time constant of the capacitor and *t* indicates the actual charging time), which indicates the completeness of the charging process. It is only possible to put the *ZT* value of ionic thermoelectric materials together with electronic ones when using them to charge the same supercapacitor. It is important to note that the correlation between *ZT* and efficiency is different when using the same material in TEGs and iTESCs. As shown in Fig. 1c, for small *n* = 0.1 (only counting the very beginning of the charging process), the charging efficiency could exceed those of TEGs, whereas, with increasing *n* (completing charging), the charging efficiency drops quickly. Moreover, if the period of Δ*T* oscillation is much longer than the charging time of the iTESCs, the device will continue to consume heat without producing additional electrical energy, thus leading to low efficiency. Optimized efficiency requires a match between the charging time and the Δ*T* oscillation period.

A more serious issue is that the charging efficiency does not describe the overall operating efficiency of iTESCs. The overall efficiency of iTESCs should include the total consumed heat *Q*_m_ and *Q*_th_ in addition to *Q*_ch_, as summarized in Equation (2):


(2) \begin{eqnarray*}
&&\eta_{{\mathrm{all}}} =\\
&&\frac{{{\mathrm{\Delta }}T\alpha _{\mathrm{i}}^2}}{{\frac{L}{C}[\rho {C}_{\mathrm{p}} + \frac{\lambda }{D}] + 2{T}_{\mathrm{H}}\alpha _{\mathrm{i}}^2 - \frac{1}{2}{\mathrm{\Delta }}T\alpha _{\mathrm{i}}^2 + \frac{{2n\lambda }}{{{\sigma }_{\mathrm{i}}}}}}.\\
\end{eqnarray*}

For typical polymer electrolytes with the highest ionic diffusion coefficients of ∼10⁻⁹ m²/s and characteristic device parameters (*L*/*C* ≈ 10⁻⁵), quantitative analysis shows that *Q*_m_ exceeds *Q*_ch_ by 10-fold, while *Q*_th_ surpasses *Q*_ch_ by four orders of magnitude. As shown in Fig. 1c, the total efficiency *η* = *E*_ch_/(*Q*_m_ + *Q*_th_ + *Q*_ch_) is orders of magnitude lower compared with the charging efficiency for the same *ZT* value. This low efficiency cannot compete with those of inorganic-based TEGs that reach 3%–7% under ambient-temperature operation. The operation of TEGs under constant Δ*T* largely reduces the contribution of *Q*_m_ and *Q*_th_. Even when comparing classic TEGs charging a supercapacitor (an equivalent electrical configuration to iTESCs), the contribution of *Q*_th_ is smaller for electronic materials due to the fast charge transport. Hence, electrolytes with *ZT*_i_ > 1 still cannot overcome this fundamental thermodynamic barrier imposed by the capacitive charging mechanism itself.

Addressing this efficiency crisis requires both incremental optimization and revolutionary innovation. Incremental approaches focus on improving existing iTESC frameworks. Enhancing ionic diffusion coefficients through liquid-crystalline electrolytes or nanoconfined channels reduces the thermal voltage establishment time, thereby lowering *Q*_th_. Reducing the thermal conductivity via polymer composites or porous structures maintains Δ*T* with less energy input. Most promisingly, dramatically increasing the electrode capacitance from supercapacitor to battery-grade levels—representing 100- to 500-fold improvement—could substantially reduce the proportion of *Q*_m_ and *Q*_th_. The challenge is to maintain the large Seebeck coefficient while enabling ion intercalation of the electrode. These strategies might elevate the efficiency to 0.1%–1%, yet cannot transcend the fundamental limitation of the capacitive charging mechanism.

Revolutionary breakthroughs demand abandoning the charge accumulation–thermal cycling paradigm to achieve continuous current generation under sustained Δ*T*. Hence, *ZT*_i_ could directly connect to the overall efficiency of devices such as TEGs. The most transformative approach involves ion–electron hybrid systems in which ionic and electronic charge carriers coexist within the same material but operate with vastly different mobilities. Recent demonstrations using highly crystalline electronic conductors (n-type perylene bisimide) have validated this concept [6]. Upon controlled oxidation in humid air, these mixed ion–electron conductors achieve ionic Seebeck coefficients of –3021 μV/K combined with electronic conductivity of 0.18 S/cm. The key distinction from iTESCs is that electrons instead of ions complete the circuit, eliminating temperature cycling. Implementation requires the rational design of crystalline organic semiconductors with tunable doping levels, exploring conducting polymer blends with optimized electronic/ionic conductivity ratios and developing 3D architectures that maximize both ionic thermodiffusion and electronic percolation pathways. Another revolutionary approach introduces reversible redox couples that are typically used in thermogalvanic cells into ionic electrolytes to enable continuous output power [7]. This strategy elevates the Seebeck coefficient of redox electrolytes that are typically 0.5–2 mV/K to >10 mV/K, thus greatly enhancing the power output. Improvement of the mass diffusion of redox species could lead to further enhancement of efficiency.

As a relatively young research field, critical conceptual clarifications of the ionic thermoelectric effect must accompany technical advances. A persistent confusion involves conflating ionic thermoelectric effects with hydrovoltaic effects—with the latter generating voltage through water-molecule adsorption/desorption at material surfaces [8]. In hygroscopic thin-film and hydrogel devices, a Δ*T* can induce water evaporation at the hot side and absorption at the cold side. This creates a water-concentration gradient, which in turn generates an ion-concentration gradient and a corresponding Nernst potential. This potential can be further modified by the Soret effect of water. Overall, the dynamics of water can generate voltages that are more than an order of magnitude larger than the intrinsic Soret effect [9] and lead to an extremely high ‘apparent ionic Seebeck coefficient’. Maintaining a clear conceptual distinction is crucial to deepening the fundamental understanding of the ionic thermoelectric effect. These phenomena exhibit distinct signatures: hydrovoltaic effects show strong humidity and environmental dependence and second-to-minute response times, while ionic thermal diffusion requires minutes to equilibrate. Rigorous systematic characterization under humidity variation, effective sealing to avoid water evaporation/absorption and time-resolved measurements could contribute to decoupling these contributions. Another confusion concerns genuine thermoelectric current versus electrode leakage from faradaic or non-faradaic side reactions. Cyclic voltammetry across electrochemical windows, long-term stability testing and inert reference electrodes provide essential validation. These clarifications are foundational to establishing scientific credibility. Meanwhile, the unique property of ionic charge carriers also offers exciting opportunities for energy harvesting. A recent work successfully integrated the two effects and demonstrated a moisture-gradient-enhanced ionic thermoelectric generator driven by both moisture and temperature gradients [10]. The device could generate energy density of 918 J m^–2^ in 1 hour that enabled the powering of wearable devices for monitoring motion and respiration.

Looking forward, the field requires strategic reorientation beyond pursuing ever-higher apparent Seebeck coefficient and *ZT*_i_ values while overlooking efficiency realities. Standardized testing protocols must report full-cycle efficiency including all energy inputs, following precedents from solar cell certification. Such protocols should specify the measurement conditions, calculation methods and required controls for fair comparison. The community must establish realistic efficiency milestones—reaching 0.1% and then 1% efficiency thresholds for viable niche applications rather than mainstream deployment. Extending ionic thermoelectrics toward sensing applications [11] can highlight intrinsic advantages such as mechanical flexibility, lightweight and biocompatibility. Key challenges lie in improving the response speed and operational stability—both fundamentally limited by low ionic mobility. In parallel, developing theoretical frameworks that link chemical composition and physical structure to large ionic Seebeck coefficients is essential for deepening understanding and guiding rational materials design. Once such foundations are established, AI-assisted machine learning may be effectively integrated to accelerate materials optimization.

In conclusion, Soret-induced thermoelectrics represents a scientifically fascinating field with impressive material achievements. The discovery that polymer electrolytes exhibit thermoelectric effects that lead to orders-of-magnitude-larger thermal voltage than electronic materials has opened up new possibilities. However, enthusiasm must be tempered with thermodynamic realism. The field’s future hinges not on incremental material optimization, but on fundamental device innovation overcoming the efficiency limitations inherent in capacitive mechanisms. Whether through ion–electron hybrid systems with controlled mobility contrast, integrated thermogalvanic cycling or as-yet-unimagined architectures, only by achieving efficiencies of >1% can Soret thermoelectrics transition from laboratory phenomena to practical technologies. The next decade will determine whether this promise materializes. Success demands honest limitation assessment, focused effort on efficiency bottlenecks and a willingness to pursue revolutionary rather than incremental advances.
